# Rethinking the politics of form: The strange case of the political novel

**DOI:** 10.12688/openreseurope.19916.1

**Published:** 2025-05-08

**Authors:** Zrinka Božić

**Affiliations:** 1 University of Zagreb Faculty of Humanities and Social Sciences, Zagreb, City of Zagreb, Croatia

**Keywords:** genre, form, novel, politics, formalism, political novel

## Abstract

The genre is an institution like a church or a university, a particular way of grouping literary works on the basis of their external and internal form, according to René Wellek and Austin Warren. But institutions are also there to be changed, and frameworks and rules can be challenged. As Fredric Jameson once observed, while literary criticism cannot do without genre, modern literary production continually and systematically undermines the concept itself. While political ideas and the political milieu dominate the political novel, according to Irving Howe, the literary form remains intact. Wellek and Warren therefore rightly question whether it is even possible to speak of a distinct genre when the grouping (of novels) is based solely on the theme and not on the form itself. The fact that Robert Boyers, one of the few authors to have dealt with the political novel in depth, ultimately abandoned the idea of a separate literary genre shows that Wellek and Warren’s observations have hit the core of the problem. So the question arises: are there other aspects besides content that make a novel political? Why does the political novel appear in so many different guises (such as utopia, dystopia, spy novel, war novel, thesis novel, proletarian novel, partisan novel, etc.)? Is this the cause of the problem, or is it simply the law of the novel as an unfinished genre in Bakhtin’s sense?

The beginning of the modern genre debate is primarily associated with Benedetto Croce’s *Aesthetic as a Science of Expression and General Linguistic* or simply *Aesthetics* ( *L’Estetica come scienza dell’espressione e linguistica generale*, 1902), in which he argues that the theory of artistic and literary genres is nothing more than an “intellectualist error” ( 1 on page 35). In his opinion, the theory of genres (when transformed into a set of rules) impoverishes artistic creation, suppresses originality and creates false standards of judgement. The only reason for retaining the concept of genre is of a pragmatic nature – the organisation of books on shelves. Or, as Croce explains:

From the theory of artistic and literary kinds derive those erroneous modes of judgement and of criticism, thanks to which, instead of asking before a work of art if it be expressive and what it expresses, whether it speak or stammer or is altogether silent, they ask if it obeys the *laws* of epic or of tragedy, of historical painting or of landscape. While making a verbal pretence of agreeing, or yielding a feigned obedience, artists have, however, really always disregarded these *laws of the kinds*. Every true work of art has violated some established kind and upset the ideas of the critics, who have thus been obliged to broaden the kinds, until finally even the broadened kind has proved too narrow. ( 1 on page 36–37)

“What Croce provides here”, Hans-Robert Jauss argues, “as an annihilating attack upon the normative genre concept once again presupposes (however unconsciously) that precise state of affairs in which the historical reality, the function (understood within the aesthetics of production), and the hermeneutic achievement of the genre concept would be demonstrable” ( 2 on page 78). Jauss thus turns the demand, which should actually be an expression of the sharpest criticism of the concept of genre, in favour of a theory of genre, and not just any theory of genre, but one that brings the historical perspective of observation back into play after the dominance of the ahistorical structuralist (formalist) approach. Even if a single literary work attempts in the most extreme way to negate all traditions, it presupposes everything that preceded it in a diachronic perspective and in relation to which its originality is to be evaluated. Therefore, according to Jauss, it would be useful to investigate how genre concepts and genre classification systems have influenced the way literature has been written and read in the past and to develop a theoretical model that explains how genre concepts work in general. Although Jauss focuses primarily on mediaeval literature (and mediaeval genre categories do not coincide with modern ones), his position is embedded in “a more general theoretical discussion which involves the appraisal of a series of modem theories of genre, ranging from Croce’s anti-generic hypothesis to the work of the Russian Formalists and Czech Structuralists” ( 3 on page 127). Jauss engages in critical dialogue and argues that a genre theory should include the function of reception, “a deficiency he seeks to remedy with his own brand of *Rezeptionsästhetik*, grounded in the versatile concept of the ‘horizon of expectation’” ( 3 on page 127).

Following Ortega y Gasset’s *History as a System* ( *Historia como sistema*, 1942) and de Saussure’s conception of language (as a system), Claudio Guillen, who advocates a structuralist approach to literary history in his study *Literature as a System*: *Essays Toward the Theory of Literary History* (1971), describes genre as a call to form that is simultaneously directed towards the future and the past. While the past refers to all works already written, the future presupposes a beginner, an apprentice, a future writer or an informed critic. For, as Guillen explains, “the conception of a particular genre may not only incite or make possible the writing of a new work; it may provoke, later on, the *critic’s search for the total form of the same work*” ( 4 on page 109). What is at stake, he argues, “is the idea of form in the tradition of Aristotle’s *Poetics* – the very context in which the European history of genre theory appears to make sense” ( 4 on page 109). As he explains, in the Aristotelian framework, form and content (or matter) are two intrinsic characteristics that define the mode of being of any object, and in the subsequent analysis “an effort of abstraction is needed in order to distinguish between the two elements that have gone into its making: the matter of which the object was made; and the form, or principle of *informing* and structuring, which made it the actual object that it is” ( 4 on page 110). With his plea for a structuralist history of literature at the beginning of the 1970s, Guillen repeats the long-ago gesture of Yuri Tynianov, who in 1924 (almost five decades earlier) enriched formalism, which was characterised by the reduction of genres to a mere repertoire of specific artistic techniques (as elaborated by Shklovsky), with an innovative theory of literary evolution. Genre shifts, Tynianov claims, for it is a dynamic system that “arises (from anomalies and embryonic ideas in other systems) and declines, turning into the rudiments of other systems” ( 5 on page 155). In other words, a genre can only be identified through a comparison or contrast with a traditional genre, which is why it cannot be conceptualised as part of a static system. As Tynianov explains, the new phenomenon displaces the old, “takes its place and, without being a ‘development’ of the old one, nevertheless acts as its substitute”; but if the substitution does not take place, the genre ceases to exist, disappears ( 5 on page 156). This makes it clear that what is unavoidable in the long tradition of literary theory and criticism since Aristotle’s *Poetics* is in fact changeable, unstable and constantly undermined in the process of literary production. This is why Fredric Jameson is right to define genres as literary institutions “or social contracts between a writer and a specific public, whose function is to specify the proper use of a particular cultural artifact” ( 6 on page 92). But just as the implicit performative is always exposed to the possibility of failure and misinterpretation, the reception of written genres that are deprived of the original performative situation cannot be controlled. Therefore, written literary genres are to a large extent “absorbed by this (impossible) attempt to devise a foolproof mechanism for the automatic exclusion of undesirable responses to a given literary utterance” ( 6 on page 93).

If we take all this into account and do not abandon the concept of genre, we must ask ourselves in the face of an ‘obvious genre’ that is both a literary fact and a social phenomenon: what is a political novel? Why is it an obvious genre? How do we deal with the political novel? At first glance, things seem very simple: it is easy to recognise a political novel, it is not difficult to find examples of political novels. But what distinguishes them from all other types of novels? What do they all have in common that we put them so clearly in the same category? Irving Howe, the author of one of the first comprehensive studies of the political novel, does not want to insist on a rigid definition of the political novel or on strict classifications, but is rather interested in observational perspectives. For Howe, neither the political nor the psychological novel is an independent genre category, since in both cases one cannot speak of fundamental differences in literary form. According to Howe, the relationship between politics and literature is not a constant, so that it is necessary to show how and why politics determines certain types of novels more strongly. In his view, a political novel is “a novel in which we take to be dominant political ideas or the political milieu, novel which permits this assumption without thereby suffering any radical distortion and, it follows, with the possibility of some analytical profit” ( 7 on page 19).

In discussing the emergence of the modern novel as a genre, Howe emphasises several of its sources (romance, pastoral, historical chronicle, early newspaper report), paying particular attention to the picaresque novel, whose protagonist, in his view, strongly anticipates the new forms of social mobility characteristic of future modern society. Inherent in a political novel are internal tensions, which is why the author, like a skilful dialectician, must be able to deal with several ideas simultaneously, recognise their contradictory and interdependent relationships and grasp the way in which they are transformed in the novel into something other than mere ideas of a political programme. Regardless of the author’s intention and ideological position, for Howe a political novel cannot be based on a political idea alone. Despite the role played by politics, literature as such should not be called into question. It is interesting to note that in 1924, the same year that Tynianov published his “Literary Fact” in LEF, a little-known study by Morris Edmund Speare was published under the title *The Political Novel*, in which the author claims that it is “a work of prose fiction which leans rather to ‘ideas’ than to ‘emotions’ […] and where the main purpose of the writer is party propaganda, public reform, or exposition of lives of the personages who maintain government, or of the forces which constitute government” ( 8 on page ix). Interestingly, René Wellek and Austin Warren, in their famous *Theory of Literature*, refer to Speare’s study when discussing the problem of genre and reject the idea of a political novel as an independent literary genre because one could then also speak of an ecclesiastical novel, a sea novel, a novel about the Oxford Movement and so on. To speak of a political novel as a literary genre, it is not enough, in their opinion, to group the novels according to content and sociological aspects ( 9 on page 1949, 242). For Speare, the political novel is an offshoot of the historical novel and the so-called novel of purpose, a specific form that developed in English and American literature in the 19th century. For Speare and Howe, however, the term novel in the phrase political novel implies almost “always a bourgeois-realist novel” ( 10 on page 3). While the “elitist social assumptions” were, according to Michael Wilding, “at the base of Speare’s narrow attempt to define a genre”, Howe broadens the perspective somewhat ( 10 on page 2). Not enough, however, to include the whole range of novels that do not fit into the framework of bourgeois realism, novels by Aldous Huxley, Yevgeny Zamyatin, George Orwell, Margaret Atwood and many others.

Almost three decades after Howe, Robert Boyers points out that the project of a political novel only makes sense if its central motif is change. Whether the political novel is radical or conservative, it is always about the projection of struggle as the positioning of objects, persons and institutions in a world that has changed and can change again. Following Louis Althusser and Jameson, Boyers claims that the concept of the absent cause, which eludes totalisation and makes it impossible to grasp and describe comprehensively, is crucial to the temporality of the novel. At the same time, nothing presented at the narrative level explains the reasons for the sequence and dynamics mentioned above. That is, the novel hints at what we should know, but paradoxically denies us this knowledge. Great political novels, Boyers argues, point to an absent cause, and the only way to grasp it is through the political unconscious, which functions as a “repository of narratives and of narrative schemas or codes” ( 11 on page 21). Echoing Jameson’s concept of the political unconscious, Boyers warns that such schemas are never available to us in their pure form: “They are unconscious in the sense that we resort to them without trying and are often unaware of how entirely we rely on them to resolve uncertainty” ( 11 on page 21). In a political novel, however, identifying the absent cause becomes a problem, as different paradigms overlap and challenge each other, making it impossible to fully resolve the doubts in the story. In the endeavour to grasp the absent cause through the aforementioned narrative schemes, it is important that the surface layer of the text does not disappear from the reader’s field of vision, Boyers emphasises. Twenty years later, the same author unfortunately argues that it is no longer “useful to speak of ‘political novels’” ( 12 on page 4). His later book, *The Dictator’s Dictation: The Politics of Novels and Novelists*, consists of a series of readings of various novels, without any serious explanation of the actual reason for not theorising the concept of genre further. Other similar books, such as Stuart Scheingold’s *The Political Novel: Re-Imagining the Twentieth Century* and David Caute’s *Politics and the Novel during the Cold War* ^13^, do not go beyond a simple listing of well-known examples of novels without additional theoretical problematisation.

However, as far as the theoretical examination of the political novel as a literary genre is concerned, the most important contribution was made in the 1980s by Susan Rubin Suleiman, who focussed primarily on the *roman à thèse*, a specific type of realist novel that has such a bad reputation that “no writer would claim it as their own” ( 14 on page xiii). In her attempt to ‘rescue’ the *roman à thèse*, Suleiman takes structuralist narratology as a starting point to shed light on the narrative techniques and mechanisms of this particular type of political novel, which is usually described as didactic without deeper theoretical insights. The story of the *roman à thèse* is, as Suleiman emphasises, teleological in nature and as such requires “an unambiguous interpretation” and “a rule of action applicable (at least virtually) to the real life of the reader” ( 14 on page 54). Regardless of whether this ambiguity of interpretation and the associated rule of action is explicitly expressed by the narrator or whether the reader can easily deduce it from the text due to clear narrative indications, it is crucial to exclude any possibility of a plural reading. How is this achieved? In her opinion, with rhetorical means through the redundancy of the discourse, whereby the “signification is excessively named”, as Roland Barthes once described it ( 15 on page 79). This kind of redundancy is typical of “the archaic – or infantile – era of modern discourse, marked by the excessive fear of failing to communicate meaning” and can be associated with the so-called readable texts ( *le texte lisible*) in general ( 15 on page 79). Accordingly, the redundancy of narrative communication reduces the amount of information conveyed and increases the possibility of understanding the message correctly. If we add the doctrinal intertext on which the novel is based (articulated either by an omniscient narrator or by one of the characters), it becomes clear that for the narrative content of the *roman à thèse* a specific story is not what matters, but “the way a story is integrated into a specific system of signification or, if one prefers, into a specific mode of discourse” ( 14 on page 63). In contrast to an ordinary realist novel, such a novel operates within a clear dualistic value system that encourages the reader to choose between these values. Suleiman uses the *roman à thèse* to show that the genre is “not a scientific formula”, but “a system of dominant traits” and the predominant feature of this type of novel is “the manifest intention to communicate an unambiguous, virtually exhortative message” ( 14 on page 243). Furthermore, Suleiman shows that form, i. e. the different types of narrative redundancy, plays a key role in the functioning of such a novel. At the same time, she lays the groundwork for an analysis of the political effects of narrative discourse that departs from its formal aspects.

The terms *genre* and *form* have often been treated as “synonyms or near-synonyms”, but as Caroline Levine shows in her groundbreaking book *Forms: Whole, Rhythm, Hierarchy, Network*, the crucial difference lies in the way they “traverse time and space” ( 16 on page 13). While the determination of the genre of an individual literary work is the result of a historically specific process of interpretation, the form stands in a completely different relationship to the context, since it organises both social and literary objects and at the same time remains stable. As a proponent of the so-called new formalism, Levine insists on extending the scope of the concept beyond the realm of aesthetics. She also claims that the history of the concept of form itself shows that art does not take precedence over it: “form can point us to visual art, music, and literature, but it belongs equally to philosophy, law, mathematics, military science and crystallography” ( 16 on page 2). Accordingly, for Levine, the concept designates “all shapes and configurations, all ordering principles, all patterns of repetition and difference” ( 16 on page 3). This means that we are surrounded by different forms and that the literary form does not stand outside the social. Rather, different forms coexist, touch each other, meet and collide, and in this clash of different orders it is sometimes difficult to predict the consequences. Compared to genre, forms show greater stability which is why they can be transferred from one context to another. As Levine explains, genres “can be defined as customary constellations of elements into historically recognizable groupings of artistic objects, bringing together forms with themes, styles, and situations of reception, while forms are organizations or arrangements that afford repetition and portability across materials and contexts” ( 16 on page 13–14).

So, what about the novel, or to be precise – the political novel? If one wants to preserve the idea of the political novel and not allow it to be absorbed into the influential notion of the inherent politics and ideology of literature that has been adopted by Marxist literary criticism (from the Frankfurt School to the post-Althusserian variants of literature as always already ideological) and other contemporary theoretical currents, one should first examine the political novel as novel, or as Mikhail Bakhtin once wrote:

a sole genre that continues to develop, that is as yet uncompleted. The forces that define it as a genre are at work before our very eyes: the birth and development of the novel as a genre takes place in the full light of the historical day. The generic skeleton of the novel is still far from having hardened, and we cannot foresee all its plastic possibilities. ( 17 on page 3)

Of all the major genres, Bakhtin continues, the novel is the only one that is younger than the book and as such is exclusively part of written culture, which is why it requires a specific kind of silent reception – reading. He also argues that “the novel has no canon of its own, as do other genres”, for “only individual examples of the novel are historically active, not a generic canon as such” ( 17 on page 3). For Bakhtin, the study of other genres resembles the study of dead languages, while the novel, as a young and living genre, requires a completely different approach. As the only genre to have emerged in a new and modern epoch of world history, the novel represents a unique literary phenomenon. While other genres entered this epoch as already fixed forms that adapted to new conditions, the novel is, in Bakhtin’s words, “a creature from an alien species” that cannot coexist with other genres ( 17 on page 4). It struggles for supremacy, and when it asserts itself, other (mostly older) genres are decomposed, deformed, parodied, undermined, displaced, and assimilated (Bakhtin uses the phrase *novelisation of genres*). Since it is a genre capable of engaging with the tendencies of what Bakhtin would call “a new world still in the making”, it is also capable of anticipating “the future development of literature as a whole” by stimulating the renewal of other genres and infecting them “with its spirit of process of inconclusiveness” ( 17 on page 7). In this way, the novel becomes the motor of change in the literary world. For this reason, it remains a constant challenge for literary history and theory. Due to such character of the novel, literary theory is unable to identify any firmly defined characteristic of the novel without at the same time undermining it. As Bakhtin illustrates:

- the novel is a multi-layered genre (although there also exist magnificent single-layered novels); the novel is a precisely plotted and dynamic genre (although there also exist novels that push to its literary limits the art of pure description; the novel is a complicated genre (although novels are mass produced as pure and frivolous entertainment like no other genre); the novel is a love story (although the greatest examples of the European novel are utterly devoid of love element); the novel is a prose genre (although there exist excellent novels in verse). ( 17 on page 9)

If a political novel is a novel, what is the problem? The novel is to the modern age what the epic was to the ancient, pre-modern world, and as such, according to Bakhtin, a specific conception of time is inherent to it. Unlike the epic world, which is separated from the time of the performer and his audience by an absolute epic distance, time and world lose their completion when the present becomes the centre of being-in-the-world ( *das in-der-Welt-sein*) in the Heideggerian sense. It is a world in which there is no absolute beginning and the end is not yet in sight. While the absolute epic past is completed and finished, the present is problematic, fragmentary, contradictory, unfinished, and so is the novel as a genre, because it was created from the beginning on the basis of a new sense of time. And when we speak of the time perspective, we cannot avoid recalling Jauss’s horizon of expectation ( *Erwartungshorizont*), which is a further development of Hans-Georg Gadamer’s earlier concept of the horizon of the hermeneutic situation ( 18 on page 301). For the horizon, as Gadamer interprets it, regulates our possibilities of interpretation, and as the end line of our seeing it sets a limit to precisely these possibilities that arise from our situation in the world. However, if we place Jauss’s horizon of expectation as a system and structure of expectations (concepts, prejudices and preconceived opinions) that the reader brings to the text in the context of thinking about the genre, we can consider the horizon of the genre tradition as determining the reception of the work.

Hence, what is the problem with political novel? It appears in various genres (serious, entertaining, fantastic, realistic, pseudo-documentary, utopian, dystopian, historical, proletarian, partisan, with thesis, without thesis, and the list never ends) precisely because it is a novel. And since one of the aims of the Caponeu project is to research and map political novels in Europe in space and time, to establish social and political coordinates of their production and reception (events, tendencies, conditions of possibility, effects), such mapping should also include the internal mapping of the political novel as a genre – its many faces and iterations. In his attempt to summarise different structural dimensions of genre, John Frow lists the following: a set of *formal features*, a *thematic structure* “which draws upon a set of highly conventional topics or *topoi*”, *a situation of address*, a more general *structure of implication* (which includes a range of relevant background information, and knowledges), a *rhetorical function* (pragmatic effects of the text), and physical *setting* “which takes on the force of a regulative *frame*” that “alerts us to the way it works (its rhetorical function), and draws our attention towards some of its features and away from others” ( 19 on page 9). These structural dimensions should, in my opinion, be taken into account when it comes to the political novel. None of the studies that have dealt explicitly with it (from Speare and Howe to Scheingold) take them as their starting point, but mostly revolve around thematic features, which is probably why a deeper problematisation and discussion is lacking. Departing from Michel Foucault’s notion of discourse (as a practice that systematically forms the objects of which it speaks), Frow speaks of the *performance of genre*. He proposes a shift away “from an ‘Aristotelian’ model of taxonomy in which a relationship of hierarchical belonging between a class and its members predominates, to a more reflexive model in which texts are thought to use or perform the genres by which they are shaped” ( 19 on page 25). Inspired by Jacques Derrida’s description of the *law of genre* ( 20 on page 230) as not-belonging but participating in one or several genres simultaneously or participating without belonging (without having a membership of a set), Frow argues that the participation “of texts in genres cannot mean a subsumption of the members of a class in the closed totality to which they belong” ( 19 on page 28). Instead, “texts work upon genres” while at the same time being shaped by them, for “genres are open-ended sets, and participation in a genre takes many different forms” ( 19 on page 28). For this reason, we should think about the politics of the political novel by starting from the concept of form and considering the above-mentioned structural features of each text as well as its genre performance.

In light of the current state of literary studies following the peak of post-structuralism and critical theory more broadly, several scholars have recently advocated for a return to formalism, each from their own perspective. In this context, Caroline Levine’s essay “Strategic Formalism: Toward a New Method in Cultural Studies” (2006), followed by her influential book *Forms: Whole, Rhythm, Hierarchy, Network* (2015), played a pivotal role. According to her, literary forms are socially and politically forceful” even though “they do not derive their power from their fit with existing or emerging patterns of social life” ( 21 on page 626). Instead, she argues that literary forms stand in a “destabilizing relation to social formations”, they often collide “with social hierarchies rather than reflecting or foreshadowing them” ( 21 on page 626). In this way, they have the ability to disrupt and reshape political relationships in unexpected, random and often confusingly chaotic ways. Hence, for Levine, politics emerges “as a set of jumbled and overlapping ways of organizing bodies, words and objects” ( 21 on page 630). This means that a single act of violence cannot be identified as political, what it requires to be regarded as such is taking part in “a series of such acts, in a context in which hierarchies repeatedly organize experience” ( 21 on page 631). In other words, if one insists on the politics of a literary text, one “implicitly abstracts from its particularity to an iterable, portable set of patterned relationships that organize the social” ( 21 on page 631). According to Levine, the form cannot be considered apolitical or ahistorical because it is simultaneously conceptual, abstract, generalising and transhistorical. It requires the full range of close reading techniques without losing sight of the social context. Levine describes this approach as a strategic formalism or a post-post-structuralist formalism that is “deconstructive in that it acknowledges the political risks of abstractions, binaries and seemingly transhistorical forms, while also recognizing that we cannot do without them” ( 21 on page 633). In this way, formalism provides an ideal set of methods for analysing the nuanced interactions between different orders and forms. However powerful and complex, Levine argues in her influential book, no one form “causes, dominates, or organizes all others” ( 16 on page 16). Literary forms thus function in their own way, and the novel as a literary form does not “simply mirror or contain prior political realities” ( 16 on page 16). Instead, it collides with other forms of the world and reorganises experience “in ways that call for unconventional political strategies” ( 16 on page 17). Levine rejects traditional ideological critique in favour of strategic political action that considers how different literary, social, political and artistic arrangements shape and influence our experience. In other words, it is not just about identifying the political novel in a single literary text, but also about examining its political implications. Simply put, it is about discussing not only what the political novel is, but also what it does, and since it occurs in different genres, it is necessary to describe how this collision comes about and what its consequences are. Today, when many literary scholars “under the banners of surface reading, postcritique, the descriptive turn, cognitive studies, and some strains of affect theory” have arguably abandoned the political aims of criticism, one has to agree with Caroline Levine and other advocates of formalism, for it has the potential to develop and discuss “ideas about making, about making relations, about making spaces and orders deliberately and justly” ( 22 on page 4). In other words: When it comes to form itself, the stakes are high.

## Ethics and consent

Ethical approval and consent were not required.

## Data Availability

No data associated with this article.
