# Supplementary material for: Rethinking the politics of form: The strange case of the political novel
Source: Open Res Eur. 2025 May 8;5:125. [Version 1] doi: 10.12688/openreseurope.19916.1 (PMC12181763; doi:10.12688/openreseurope.19916.1)
Supplement: Peer review history

# Reviewer report: Reviewer response for version 1

**DOI:** 10.21956/openreseurope.21544.r54915

**Published:** 2025-06-30

**Authors:** Mayte Donstrup

**Affiliations:** 1 Universidad Rey Juan Carlos, Madrid, Spain

Without a doubt, we are dealing with a very suggestive and interesting text, which leads to intense debates about the genre. However, I would like to highlight some aspects and provide some comments so that you may improve your paper.

 The text engages with classic authors as Bakhtin or Jauss. That's good. However, it would be valuable to address more contemporary discussion about the conventions and limits of the genre (from reception studies, for example). The lack of more contemporary authors and texts should be fixed (with less than 5 years).

 It would maybe be a good idea to try to follow a more conventional structure for the article (Intro, Lit Review, Methodology, Results and Discussion) so as to frame all the information here in a more cohesive and organized way.

 It is essential for the author to clearly articulate the significance of their work and delineate how it advances the current state of research. The article can potentially make a more substantial impact by addressing these concerns.

If any, are all the source data and materials underlying the results available?

Yes

Is the work original in terms of material and argument?

Yes

Is the work clearly and cogently presented?

Yes

Is the argument persuasive and supported by evidence?

Yes

Does the research article contribute to the cultural, historical, social understanding of the field?

Yes

Does it sufficiently engage with relevant methodologies and secondary literature on the topic?

Yes

Reviewer Expertise:

Media studies; political communication; reception studies

I confirm that I have read this submission and believe that I have an appropriate level of expertise to confirm that it is of an acceptable scientific standard, however I have significant reservations, as outlined above.

# Reviewer report: Reviewer response for version 1

**DOI:** 10.21956/openreseurope.21544.r54912

**Published:** 2025-06-20

**Authors:** Piotr Walewicz

**Affiliations:** 1 Kazimierz Wielki University, Bydgoszcz, Poland

The article is a critical theoretical discussion that transcends disciplinary boundaries by extending beyond literary studies and into the realm of (critical) political science. This is a very welcome and both scientifically and politically important move that aligns with the nature of contemporary discourse in social science and humanities.

 The author suggests a shift from the traditional/rigid/content-based definitions of political novel towards a more dynamic and form-centric approach, primarily inspired by Mikhail Bakhtin and Caroline Levine. They attempt to problematize what might otherwise seem an obvious literary category by revealing its nuanced nature that eludes straightforward definitions. 

 The strengths of the article are:

- a coherent logical flow, which transforms it from a simple descriptive survey into a robust theoretical intervention that invites the reader to accompany the author in their fundamental reconceptualization of the genre;
- a comprehensive literature review that includes numerous influential theorists and navigates their arguments in an understandable manner;
- juxtaposing and/or synthesizing these distinct theoretical perspectives (Croce vs Jauss, Jameson vs Boyers etc.);
- a strong case for the “strategic formalism” as an analytical framework for studying the political novel;
- using language that is both academic and accessible;
- likely serves as a strong theoretical and methodological underpinning for an ongoing scientific project that aligns with its goals of aiming to “assess the political novel as an important element of European political, social and cultural heritage” and setting out to “examine how people in different national and cultural contexts engage with contemporary political issues and thereby have their share in shaping European societies and politics” (from the CAPONEU project website);
- the potential for transdisciplinary influence on knowledge production, specifically the engagement with critical political science that could benefit the latter in both its explanatory and didactic aspects.

With that in mind, the article does have some weaknesses and/or areas for improvement, which are:

- a lack of clear, explicit thesis statement which forces the reader to infer it on their own, and the author’s use of questions to start specific arguments within the body of the article diminishes their impact if no explicit answers are given;
- an argumentative trajectory that sometimes is unclear and becomes fully understandable only upon subsequent readings of the text, e.g. some sections read more like detailed literature reviews than a clearly flowing argumentation, while the introduction goes straight into setting up the historical context of the genre debate without stating how the author specifically intends to solve their specific problem;
- the strong dependence on existing theoretical perspectives or their critiques without developing the author’s own position on the political novel - the article could use a proper conclusion where the author presents their own working model or definition rather than ending with a short presentation of the “strategic formalism” which is mentioned neither in the title nor in the abstract of the article;
- from the perspective of political science, the author presents only limited engagement with the “political” part of “political novel”, as well as the “politics” part of the “politics of form”;
- all of the above coalesce into the impression that the article is more a part of an extensive theoretical introduction to something bigger rather than a distinct, stand-alone proposition with concrete practical applications - the author should make a more explicit connection between their theoretical discussion and the CAPONEU project (which right now is limited to a single mention in the latter parts of the article);
- the whole argument is rather Eurocentric, which the author does not acknowledge, much less justifies, but this problem would go away with the inclusion of the above connection to the specific scientific project centered on European political novel.

In summary, while the article demonstrates undeniable scientific value and its goal becomes clear when taken into the larger context of the project it is a part of, the author is still encouraged to make some minor revisions for the article to serve as a self-contained and actionable theoretical proposition. These are: strengthening the thesis statement and enhancing the concluding synthesis, developing the author’s own conceptualization, deepening the analysis of what is considered “political”, providing example applications of “strategic formalism”, strengthening the link to the CAPONEU project (or providing a parallel, independent empirical association) .

If any, are all the source data and materials underlying the results available?

No source data required

Is the work original in terms of material and argument?

Yes

Is the work clearly and cogently presented?

Partly

Is the argument persuasive and supported by evidence?

Yes

Does the research article contribute to the cultural, historical, social understanding of the field?

Yes

Does it sufficiently engage with relevant methodologies and secondary literature on the topic?

Yes

Reviewer Expertise:

political science, political theory, discourse analysis, narratology

I confirm that I have read this submission and believe that I have an appropriate level of expertise to confirm that it is of an acceptable scientific standard.

# Reviewer report: Reviewer response for version 1

**DOI:** 10.21956/openreseurope.21544.r54478

**Published:** 2025-06-20

**Authors:** André P. DeBattista

**Affiliations:** 1 University of Malta, Msida, Malta

This Research Article argues that the "modern genre" debate begins with Benedetto Croce's "L’Estetica come scienza dell’espressione e linguistica generale" (1902) which argued that the theory of genres, "impoverishes artistic creation, suppresses originality and creates false standards of judgement." The ensuing debate in this Research Article is interesting and pertinent to what the author seeks to achieve in terms of discussing the genre of the political novel and the politics of form. In the same paper, the author also distinguishes between the terms genre and form, citing Caroline Levine and arguing that " While the determination of the genre of an individual literary work is the result of a historically specific process of interpretation, the form stands in a completely different relationship to the context, since it organises both social and literary objects and at the same time remains stable."

 In terms of the theoretical examination of the political novel as a literary genre, the author places some emphasis on the work of Susan Rubin Suleiman who focussed on the "roman à thèse" - a political novel "described as didactic without deeper theoretical insights" and requiring "“an unambiguous interpretation” and “a rule of action applicable (at least virtually) to the real life of the reader”".

 The author cogently argues that the political novel should be examined as a novel rather than allow it to be "be absorbed into the influential notion of the inherent politics and ideology of literature that has been adopted by Marxist literary criticism... and other contemporary theoretical currents." She suggests that the structural dimensions outlined by John Frow should be utilised when mapping the political novel. 

 Overall, this research paper presents a nuanced view of the different theoretical discussions in place while making a case for the unique case of the political novel. It is both engaging and relevant in terms of the ongoing debates on this subject.

If any, are all the source data and materials underlying the results available?

No source data required

Is the work original in terms of material and argument?

Yes

Is the work clearly and cogently presented?

Yes

Is the argument persuasive and supported by evidence?

Yes

Does the research article contribute to the cultural, historical, social understanding of the field?

Yes

Does it sufficiently engage with relevant methodologies and secondary literature on the topic?

Yes

Reviewer Expertise:

Politics, Political Science, Politics and Literature

I confirm that I have read this submission and believe that I have an appropriate level of expertise to confirm that it is of an acceptable scientific standard.
